# Comparison of three types of laser optical disdrometers under natural rainfall conditions

**DOI:** 10.1080/02626667.2019.1709641

**Published:** 2020-01-21

**Authors:** Lisbeth Lolk Johannsen, Nives Zambon, Peter Strauss, Tomas Dostal, Martin Neumann, David Zumr, Thomas A. Cochrane, Günter Blöschl, Andreas Klik

**Affiliations:** a Department of Water, Atmosphere and Environment, University of Natural Resources and Life Sciences, Vienna, Austria; b Institute of Land and Water Management Research, Petzenkirchen, Austria; c Faculty of Civil Engineering, Czech Technical University in Prague, Prague, Czech Republic; d Department of Civil and Natural Resources Engineering, University of Canterbury, Christchurch, New Zealand; e Institute of Hydraulic Engineering and Water Resources Management, Vienna University of Technology, Vienna, Austria

**Keywords:** optical disdrometer, rainfall kinetic energy, rainfall erosivity, drop size distribution, soil erosion

## Abstract

Optical disdrometers can be used to estimate rainfall erosivity; however, the relative accuracy of different disdrometers is unclear. This study compared three types of optical laser-based disdrometers to quantify differences in measured rainfall characteristics and to develop correction factors for kinetic energy (KE). Two identical PWS100 (Campbell Scientific), one Laser Precipitation Monitor (Thies Clima) and a first-generation Parsivel (OTT) were collocated with a weighing rain gauge (OTT Pluvio^2^) at a site in Austria. All disdrometers underestimated total rainfall compared to the rain gauge with relative biases from 2% to 29%. Differences in drop size distribution and velocity resulted in different KE estimates. By applying a linear regression to the KE–intensity relationship of each disdrometer, a correction factor for KE between the disdrometers was developed. This factor ranged from 1.15 to 1.36 and allowed comparison of KE between different disdrometer types despite differences in measured drop size and velocity.

## 1 Introduction

Accurate and comparable rainfall measurements are important in many fields of hydrological research in order to ensure detailed knowledge of rainfall characteristics. Early measurement methods included the stain method, the flour pellet method, oil immersion, and photographic methods (Bentley 1904, Marshall and Palmer 1948, Gunn and Kinzer 1949, Jones 1959). More recently, automated methods were developed such as impact disdrometers (Joss and Waldvogel 1967) and optical disdrometers (Hauser *et al*. 1984, Löffler-Mang and Joss 2000, Kruger and Krajewski 2002, Ellis *et al*. 2006), which are capable of continuous measurements. Optical laser-based disdrometers use laser beams to measure the number, size and velocity of raindrops. The measurement of the drop size distribution (DSD) and velocity enables the estimation of rainfall characteristics such as the accumulated rainfall amount (*R*), intensity (*I*) and kinetic energy (KE). The initial detachment of soil particles through splash erosion is dependent on the KE of the raindrops. Therefore, the ability of the disdrometers to measure the number, size and velocity of the falling drops accurately is essential in soil erosion studies.

Several authors have used disdrometers for developing KE–*I* relationships and to estimate soil erosion risk in a particular climate (Assouline 2009, Petan *et al*. 2010, Angulo-Martínez and Barros 2015, Angulo-Martínez *et al*. 2016, Carollo *et al*. 2017). However, differences in instrument design and data processing hamper the direct comparison of rainfall data from different types of disdrometers. Differences such as instrument rainfall resolution, measuring area, binning of drop sizes and velocities, software set-up and internal correction processes of drop measurement all affect the estimation of drop size and velocity and thus the calculation of KE. Consequently, the KE will be device-specific and it is therefore important to quantify the discrepancies between disdrometers, when using disdrometer data in rainfall erosivity estimation. Ideally, when comparing KE of different disdrometers across multiple locations, these device-specific biases in KE should be removed in order to identify spatial variations in the rainfall characteristics.

Rainfall measurements by disdrometers under natural rainfall conditions can be influenced by several factors, which can lead to erroneous measurements. Wind can affect the direction, speed and fall trajectory through the laser beam (Nešpor *et al*. 2000, Montero-Martínez and García-García 2016). As the laser beams only measure the vertical component of the falling drops, an altered angle of the fall direction due to wind can lead to inaccurate velocity estimates. Drops falling at the edge of the laser beams and their size therefore not being fully measured can cause them to be erroneously categorized. Two drops falling through the laser beam at the same time, and thus being detected as one, will lead to an overestimation of drop size. At high rainfall intensities, these effects may be more important than at lower intensities (Kathiravelu *et al*. 2016, Angulo-Martínez *et al*. 2018).

Studies comparing different rainfall measurement devices highlight the challenges of accurate rainfall measurement (Tokay *et al*. 2001, Lanza and Vuerich 2009, Michaelides *et al*. 2009, Liu *et al*. 2013, Montero-Martínez *et al*. 2016). Differences can even be found between instruments of the same type at the same site. Using 14 collocated Parsivel disdrometers Tapiador *et al*. (2017) found that a single disdrometer may underestimate rain intensity by up to 70% due to the limited measuring area. The need for a correction method making use of a reference instrument is therefore crucial. This can be achieved by collocating disdrometers with rain gauges or other disdrometers to quantify measurement uncertainty and biases. Especially the Thies Clima LPM and the OTT Parsivel (versions 1 and 2) disdrometers have often been the subject of inter-comparison with other devices (Krajewski *et al*. 2006, Frasson *et al*. 2011, Tokay *et al*. 2013, Raupach and Berne 2015, Park *et al*. 2017, Angulo-Martínez *et al*. 2018). The studies found discrepancies between the devices to a varying degree depending on the investigated instruments. Investigated parameters include rainfall amount, intensity, drop size and velocity distribution, drop concentrations, KE and radar reflectivity. The measurement of these rainfall characteristics was shown to be influenced by rainfall intensity (Krajewski *et al*. 2006, Tokay *et al*. 2014, Park *et al*. 2017), as well as the design and data processing of the disdrometers (Frasson *et al*. 2011, Tokay *et al*. 2013, Angulo-Martínez and Barros 2015). By applying various data filters and correction schemes to the DSD, some studies have managed to reduce the differences between instruments (Raupach and Berne 2015, Angulo-Martínez *et al*. 2018).

The abovementioned comparative studies have collected varying amounts of data under different climates at various locations (Australia, France, Mexico, South Korea, Spain, Switzerland, the USA). Data from central Europe (i.e. Austria) does not seem to be available in the literature. Additionally, the effect of differences between disdrometers on KE has been studied as one of the parameters calculated from the DSD, but it has rarely been the main focus in comparative studies. While comparisons of Parsivel and Thies seem to be rather extensive, the Campbell PWS100 has rarely been investigated (Gires *et al*. 2016, Montero-Martínez *et al*. 2016), and to the best knowledge of the authors, a comparison of the Campbell, Thies and Parsivel disdrometers has not been published. Therefore, the aim of this study is to compare these three types of disdrometers placed at the same site under natural rainfall conditions in order to quantify the differences in measured rainfall, drop size and velocity. Furthermore, to facilitate the comparison of KE between different types of disdrometers, a method for correcting KE for each disdrometer is proposed.

## 2 Materials and methods

**Figure 1.**
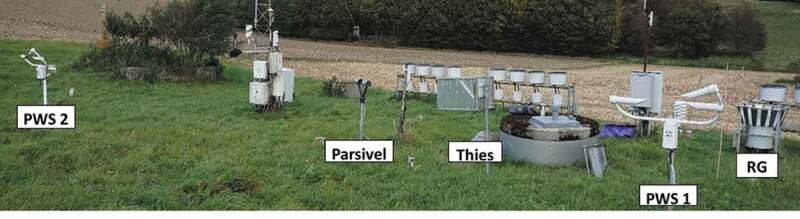
Experimental set-up of disdrometers and rain gauge at the HOAL site.

Measurements for all devices were carried out from August to October 2018 and from March to May 2019 in the Hydrological Open Air Laboratory (HOAL) Petzenkirchen, in Lower Austria (Blöschl *et al*. 2016). The long-term (1990–2014) mean annual precipitation and temperature at the site are 823 mm and 9.5°C. All devices were placed at the same measuring height of 1 m above ground and the laser heads were oriented in the north–south direction (Fig. 1). The precipitation gauge OTT Pluvio^2^ by OTT Messtechnik was used as a reference for the rainfall measurement. The rain gauge (RG) applies a weighing principle and outputs rainfall per minute. The disdrometers investigated include the Present Weather Sensor PWS100 by Campbell Scientific (PWS), the Laser Precipitation Monitor by Adolf Thies (Thies) and the Present Weather Sensor OTT Parsivel (version 1) by OTT Messtechnik (Parsivel). Two identical disdrometers of the type PWS100 were used, which are referred to as PWS 1 and PWS 2. Results from PWS 1 were only available for the measurement period in 2018. The specifications of the devices are given in Table 1. All investigated disdrometers are laser-based optical devices, and they record the rainfall characteristics at 1-min intervals. The measuring area of the devices ranges from 40 to 54 cm^2^. The devices monitor the falling raindrops going through a laser beam and are, thus able to classify the drops by size and velocity. The PWS has 34 drop size classes and 34 velocity classes, Thies has 22 drop size classes and 20 velocity classes, while Parsivel has 32 classes for both drop size and velocity. The drop size and velocity classes and their widths differ between the disdrometer types. The PWS has the same classes for drop size and velocity. Thies and Parsivel have the drop size classes divided independently from the velocity classes. For all disdrometers, the width of each drop size and velocity class increases with increasing size and velocity. The resulting matrix of drop sizes and velocities can be used to calculate the kinetic energy KE (J m^−2^) of the rainfall per minute by

(1) $$KE=\sum N_{i,j}\cdot \frac{1}{12\cdot A}\cdot \pi \cdot \rho \cdot 10^{-6}\cdot {D_{i}}^{3}\cdot {v_{j}}^{2}$$

**Table 1.** Specifications of the rainfall measurement devices used in this study.

Device	Measurement method	Measuring area (cm^2^)	Rainfall resolution (mm)	Intensity range(mm h^−1^)	No. of drop size and velocity classes (range)
Precipitation gauge OTT Pluvio^2^	Balance principle	400	0.01	6–1800	-
PWS100 Present Weather Sensor (Campbell Scientific)	Laser-based sensor, 830 nm	40	0.0001	0–400	34 (0.1–30 mm) 34 (0.16–30 m s^−1^)
Laser Precipitation Monitor (Adolf Thies)	Laser-based sensor, 785 nm	44.1	0.01	<0.005–1000	22 (0.16–>8 mm) 20 (0.2–20 m s^−1^)
Present Weather Sensor Parsivel (OTT Messtechnik)	Laser-based sensor, 650 nm	54	0.01	0.001–1200	32 (0.2–25 mm) 32 (0.2–20 m s^−1^)

where *N_i,j_* is the number of detected raindrops in a size class *i* and velocity class *j, A* is the measuring area of the disdrometer (m^2^), *ρ* is the density of water (g cm^−3^), *D_i_* is the mean drop diameter (mm) of size class *i* and *v_j_* is the mean fall velocity (m s^−1^) of velocity class *j*.

The PWS disdrometer operates on a different principle from the Thies and Parsivel disdrometers. The PWS disdrometer has four horizontal light sheets parallel to each other. When a drop falls through the light sheets, the light is scattered and detected by the two angled detectors with some time delay, which enables the calculation of the drop fall velocity and drop size (Ellis *et al*. 2006, Campbell Scientific Inc. 2012). Thies and Parsivel both operate based on the principle of light occlusion. The transmitter produces a single horizontal sheet of light going to the receiver. When a drop passes through the laser beam the output voltage is blocked according to the diameter of the drop, whereby the size of the drop can be determined. Velocity is determined from the duration of the drop entering the light sheet to it leaving it again (Löffler-Mang and Joss 2000, OTT 2005, Thies Clima 2015).

All disdrometers apply some sort of correction of the rainfall measurement within their internal programming, although the manuals are not very clear about the corrections made. In the internal PWS calculations, it is assumed that the drops are distorted similarly when they fall (Campbell Scientific Inc. 2012). For Parsivel it is stated that a correction is made for the oblateness of larger drops (Löffler-Mang and Joss 2000, Battaglia *et al*. 2010) and that it recognizes edge events, when drops only partially intersect the measuring area (OTT 2005). The Thies manual simply states that the particles are checked for plausibility such as edge hits. The PWS also outputs the number of error and unknown particles. Error particles have not passed the quality checks and are discarded (e.g. drops falling through the edge of the measurement area). Unknown particles could either not be correctly classified within the system of the disdrometer or they fall outside the natural behaviour of drops. Two overlapping particles are also classified as unknown. Drop size and velocity measurements of unknown particles are excluded from the matrix, but a correction is made within the output of intensity and precipitation amount measurements by adding one particle with the characteristics of the average particle falling at the time of detection (Campbell Scientific Inc. 2012).

Further features to minimize erroneous rainfall measurements include the sensor heads on the Parsivel being equipped with a splash protection in order to prevent splashing from the housing of the disdrometer (OTT 2005). The Thies disdrometer had a wind protection element mounted around it during the events in 2018, to reduce the swirling of drops before they enter the measuring area (Thies Clima 2019). The mean wind speed of each event was recorded by a weather station, but the effect of wind was not investigated further in this study. All disdrometers are also capable of classifying the type of precipitation into classes such as drizzle, rain, snow or hail. This classification of precipitation type was used to exclude time intervals when snow was detected.

For the analysis of the rainfall measurements by the three types of disdrometers, eight rainfall events with a total rainfall amount above 5 mm were selected. To be identified as an event, it had to be separated from the previous event by a dry period of more than 6 h. For the eight events analysed in detail, the length of each event was set to the same number of minutes for each device to enhance comparability. For each event, the maximum 30-min intensity (max *I*_30_) and the mean intensity was calculated from the rain gauge data.

Where not otherwise stated, all data, including rainfall amount and intensity, were taken directly from the output of the disdrometers. Additionally, to check this output, the rainfall amount, *R* (mm), was calculated from the DSD by

(2) $$R=\frac{4}{3}\pi \sum \left(\frac{1}{A}N_{i,j}{\left(\frac{D_{i}}{2}\right)}^{3}\right)$$

where *A* is the measuring area of the disdrometer in mm^2^, *N_i,j_* is the number of drops in size class *i* and velocity class *j* and *D_i_* is the mean diameter of drop size class *i*.

The following statistical performance parameters were used to quantify the differences in rainfall measurement between the devices. The percentage error, *E*, between the disdrometer values and the reference value of the RG was calculated as

(3) $$E=\frac{R_{D}-R_{RG}}{R_{RG}}\cdot 100\%$$

where *R*_D_ is the rainfall amount measured by the disdrometer and *R*_RG_ is the rainfall amount measured by the rain gauge. The root mean squared error, *E*_RMS_, was estimated for the sum of all events measured by each disdrometer as compared to the rain gauge:

(4) $$E_{RMS}=\sqrt{\frac{\sum^{{\left(R_{D}-R_{RG}\right)}^{2}}}{n}}$$

where *n* is the number of events. Bias was calculated as the mean of the event differences in rainfall or KE between instruments. Relative bias was calculated as the median of the percentage error in rainfall or KE between devices calculated for each event as shown in Eq. (3).

In addition to the event analyses in the HOAL, longer term comparisons of the performances of the devices were conducted. The number of compared days ranged from 60 to 256. PWS 2 and Parsivel were both placed at the HOAL Petzenkirchen site and they were therefore compared to the same OTT Pluvio^2^ as in the event analysis. PWS 1 was placed at a site in Mistelbach, Lower Austria and was compared with a standard tipping bucket rain gauge. Thies was placed in Prague, Czech Republic and was compared with a tipping bucket rain gauge.

## 3 Results and discussion

### 3.1 Rainfall amount and intensity

The rain gauge recorded a total of 32 events during the measurement period. Most were small events of only a few millimetres of rain. For further analysis, eight events with a total rainfall amount above 5 mm were selected (Table 2). The total rainfall of these events measured by the rain gauge was 106.8 mm. The total rainfall measured by the disdrometers was smaller than that with a percentage error of −3% for PWS 1 (only the five events in 2018 with a sum of 49.3 mm for the RG), –14% for PWS 2, –20% for Thies and –30% for Parsivel. The individual events give similar bias patterns. PWS 1 was always closest to the RG with a percentage error between −7.5% and 4.9%, followed by PWS 2 with –19.7% to –11.0%. Thies had a percentage error between –31.6% and –2.8% and Parsivel measured between –32.1% and –21.3% less rainfall per event. Thies measured higher total rainfall than PWS 2 only for event number 6. This trend in accumulated rainfall measurement is also reflected in the *E*_RMS_, bias and relative bias for all events presented in Table 2. All three parameters show the smallest deviation to the RG for PWS 1, followed by PWS 2, Thies and Parsivel having the largest deviation.

**Table 2.** Eight selected events and the accumulated rainfall, *R*, as measured by each rainfall measurement device and the percentage error, *E*, compared to the rain gauge, as well as *E*_RMS_, bias and relative bias for all events as compared to the rain gauge. Max *I*_30_ and mean *I* were calculated from the rain gauge measurements.

	Event 01 September 2018		Event 2 04 September 2018		Event 3 21 September 2018		Event 4 23 September 2018		Event 5 01 October 2018		Event 6 14 March 2019		Event 7 30 April 2019		Event 8 03 May 2019		All events		
Duration (h)	25.7		9.2		8.4		4.5		20.7		46.7		12.3		17.8				
Max I_30_ (mm h^−1^)	10.0		4.8		4.0		3.2		3.6		2.4		6.1		3.3				
Mean I (mm h^−1^)	0.82		0.68		0.73		1.27		0.48		0.47		1.98		0.64				
Mean wind speed (m s^−1^)	1.4		2.0		2.0		3.1		2.3		3.4		3.1		2.1				
Device	*R* (mm)	*E* (%)	*R* (mm)	*E* (%)	*R* (mm)	*E* (%)	*R* (mm)	*E* (%)	*R* (mm)	*E* (%)	*R* (mm)	*E* (%)	*R* (mm)	*E* (%)	*R* (mm)	*E* (%)	*E*_RMS_ (mm)	Bias (mm)	R. bias (%)
RG	21.2	-	6.3	-	6.1	-	5.7	-	9.9	-	21.8	-	24.3	-	11.5	-	-	-	-
PWS 1	19.6	-7.5	6.2	-1.6	6.4	4.9	5.9	3.5	9.4	-5.1	-	-	-	-	-	-	0.8	-0.3	-1.6
PWS 2	17.2	-18.9	5.3	-15.9	4.9	-19.7	5.0	-12.3	8.8	-11.1	18.8	-13.9	21.4	-12.1	10.2	-11.0	2.2	-1.9	-13.1
Thies	16.0	-24.5	4.8	-23.8	4.5	-26.2	3.9	-31.6	7.2	-27.3	21.2	-2.8	18.0	-26.0	9.7	-15.1	3.3	-2.7	-25.3
Parsivel	14.4	-32.1	4.6	-27.0	4.8	-21.3	4.2	-26.3	7.0	-29.3	15.1	-30.5	16.6	-31.9	8.1	-29.3	4.7	-4.0	-29.3

When calculating the accumulated rainfall from the DSD using Equation (2), the total rainfall amount measured by Thies increased by 17%, whereas it decreased by 5–6% for PWS 1 and PWS 2 and by 1% for Parsivel, as compared to the total rainfall given by the output of the instruments. The smaller rainfall amount estimated from the DSD of the PWS disdrometers can be attributed to the number of unknown drops. As described earlier, drops that could not be correctly classified by the PWS, but were measured, are grouped as unknown, left out of the matrix, but added to the output rainfall with the characteristics of an average drop. During all five events in 2018, PWS 1 registered 2823 error particles and 4601 unknown particles. PWS 2 registered 5353 error particles and 8966 unknown particles. This means that PWS 2 had almost twice as many error and unknown particles registered as PWS 1. The higher number of error and unknown particles of PWS 2 could contribute to the difference in the measured rainfall amount. Drops that are erroneously measured and/or discarded decrease the measured drop mass and thereby the accuracy of measurement. The reason for PWS 2 measuring more error and unknown drops is less clear. Contrary to the findings of this study, Angulo-Martínez *et al*. (2018) found a small decrease in rainfall amount from Thies, when calculating it from the DSD as compared to the output. Similarly, the reason for the increase in rainfall calculated from the DSD of the Thies disdrometer is unknown, as the manual does not give much information about any correction of rainfall accumulation. However, using the DSD to calculate total rainfall seems to greatly improve the performance of the Thies. There is not much difference between the rainfall amount from the output and from the DSD of the Parsivel. Calculating accumulated rainfall from the matrix of drop sizes and velocities has to be done with the understanding that it may not account for all of the rainfall, because the matrix may not include all drops, such as in the case of the PWS. On the other hand, for Thies (and Parsivel) it seems that the rainfall output may not include all the rainfall represented in the DSD. Per event the difference in accumulated rainfall between the output and that calculated from the DSD was rather small; however, over time, the accumulated difference can be significant. This difference of rainfall estimated from the DSD and the output of the instruments may be related to the internal corrections, but as the full extent of this internal process is not known to the user, we chose to use the given rainfall outputs of the devices in the further analyses below.

**Figure 2.**
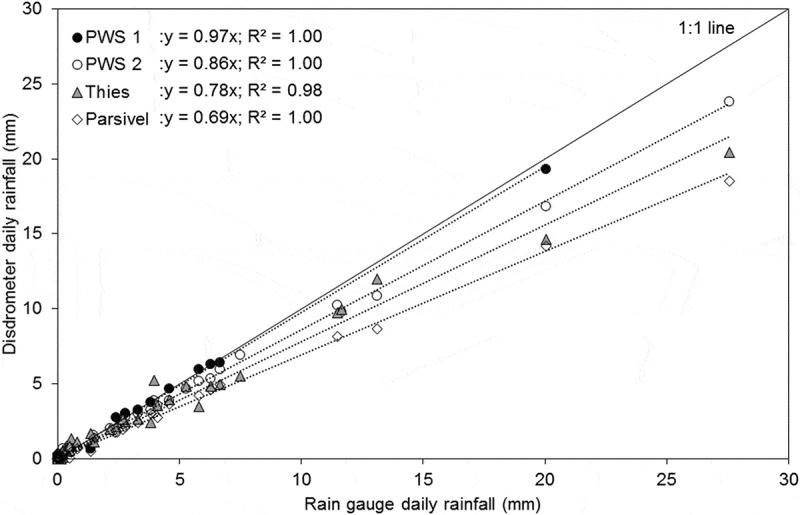
Comparison of the daily rainfall amount measured by the rain gauge and each disdrometer at the HOAL site for the measurement periods August–October 2018 and March–May 2019.

In addition to the eight events, all wet days within the observation periods were analysed. The results showed that all disdrometers tend to underestimate the rainfall accumulation compared to the rain gauge (Fig. 2). This is the same pattern as observed per event. It is also important to note, that for higher rainfall amounts the agreement between RG and disdrometers was lower than for smaller amounts.

**Figure 3.**
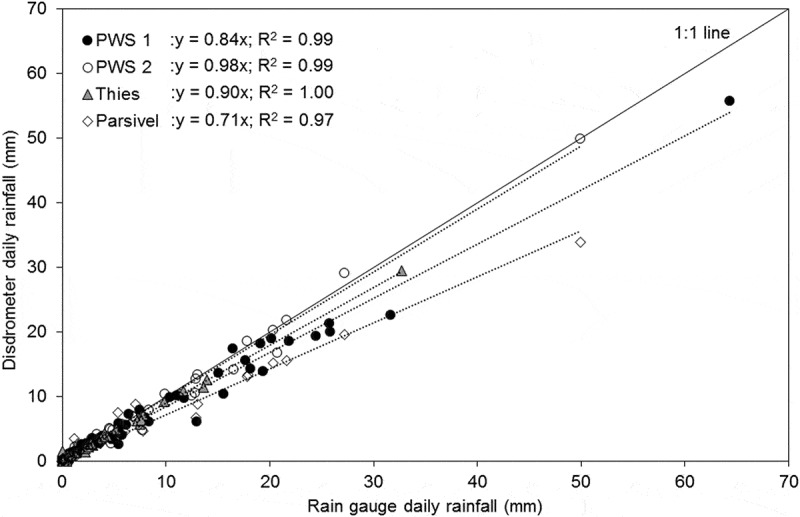
Long-term daily rainfall measurement comparison for all disdrometers at different sites (HOAL, Mistelbach, Prague).

The long-term comparison of daily rainfall between rain gauges and disdrometers at different sites (HOAL, Mistelbach, Prague) showed a very good agreement of PWS 2 with the rain gauge with a 2% overall deviation (Fig. 3). Thies measured 10% less than the rain gauge, PWS 1 measured 16% less, and Parsivel measured 29% less on average. PWS 2 and Parsivel were located at the HOAL Petzenkirchen site and were therefore compared to the same rain gauge as in the event analyses. PWS 1 and Thies were located at two other sites, which used tipping bucket rain gauges as reference. Tipping bucket rain gauges may be less accurate than weighing type rain gauges (Lanza and Vuerich 2009, Michaelides *et al*. 2009), which may affect the results for PWS 1 and Thies.

The rain gauge measurements were taken as the reference in this study; however, rain gauges are also subject to measurement errors. Therefore, deviations between the RG and the disdrometers will not only be due to measurement errors of the disdrometers. Contrary to this study, Montero-Martínez *et al*. (2016) found that the PWS100 measured higher rainfall amounts than the reference tipping bucket. In our study, this was only the case for PWS 1 for two events, where it measured 3.5% and 4.9% more accumulated rainfall. The total rainfall accuracy as stated by the manufacturer is 10%. The reason for the higher accuracy of the PWS 2 in the long-term measurements may be a result of the number of events or the type of rainfall events, although this has not been investigated further.

**Figure 4.**
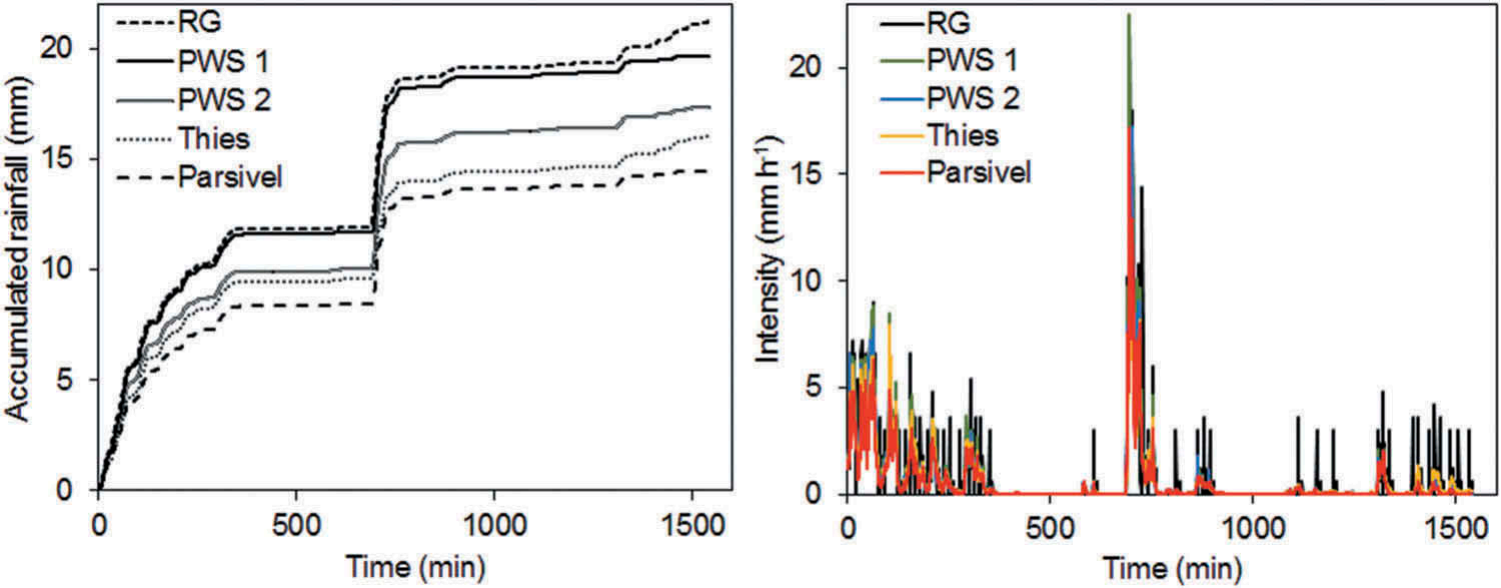
Accumulated rainfall (left) and intensity (right) over time for the event on 1 September 2018.

A large event with 21.2 mm rainfall within almost 26 h occurred on 1 September 2018. The accumulation of rainfall over time and the intensity recorded by each device is shown in Fig. 4. The temporal distribution of rainfall is similar for all disdrometers and the RG, although the total accumulated rainfall amounts of the event differ. As seen from the measured intensities over the course of the event, the devices detected rainfall at the same time. However, the rain gauge measured larger peaks of intensities, whereas especially PWS 2, Thies and Parsivel recorded less intense rainfall. Particularly at the lower intensities at the end of the event, the disdrometers did not measure the same peaks of intensity as the RG.

**Figure 5.**
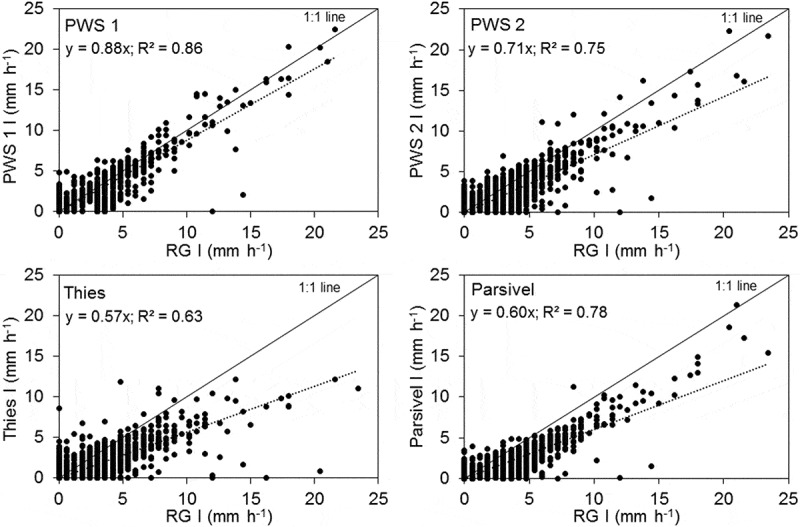
Comparison of the intensities measured per minute by the rain gauge and each disdrometer for all events.

A comparison of the measured intensities of each disdrometer compared to the rain gauge shows a large scatter of the 1-min intensity points for all events (Fig. 5). The difference between disdrometer and RG increases with increasing intensities, although this trend is not pronounced for PWS 1. All disdrometers measure less intense rainfall than the RG at higher intensities. Thies does not measure intensities above 12 mm h^−1^. Thies has the most scatter and the worst overall agreement with the RG, followed by Parsivel, PWS 2 and PWS 1. The large variability may be a result of the difference in the sampling area of the RG and disdrometers, as well as the natural variability of the rainfall, but it also indicates considerable device-specific effects (Krajewski *et al*. 2006).

### 3.2 Drop size and velocity distribution

**Figure 6.**
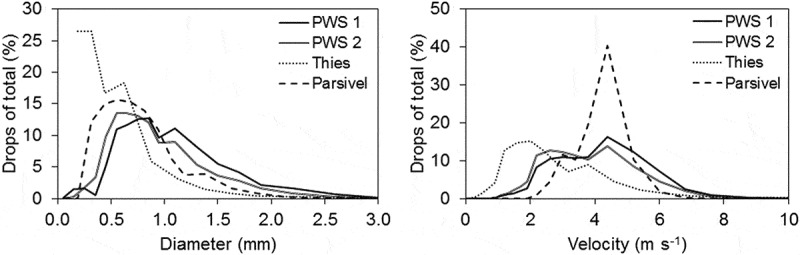
Mean drop size (left) and velocity (right) distribution of the selected events analysed. Each drop size and velocity class is shown as the percentage of drops within this class out of the total number of drops.

Figure 6 shows the DSD of all events analysed as the number of drops in each diameter size class out of the total number of recorded drops for each disdrometer. For all measured events, drop sizes between 0 and 1 mm make up the majority of the drops measured by PWS 1 (66%), PWS 2 (76%), Thies (94%) and Parsivel (83%). PWS 1 and 2 follow a somewhat similar distribution with a higher percentage (31% and 22%) of larger drops from 1 to 2 mm than Thies (6%) and Parsivel (16%). The velocity distribution as shown in Fig. 6 also indicates clear differences between the disdrometers. PWS 1 and 2 again have similar distributions with the majority of drops (52% and 58%) falling between 2 and 4 m s^−1^, whereas Thies recorded the highest percentage of drops (64%) below 2 m s^−1^ and Parsivel had the highest percentage of drops (70%) in the range of 4–6 m s^−1^. This is also reflected in the mean and median drop sizes and velocities. Table 3 shows that Parsivel measured a slightly lower median drop size of 0.7 mm compared to that of 0.9 and 0.8 mm measured by PWS 1 and 2, respectively, and slightly higher mean and median velocities. PWS 1 and 2 measured a similar number of drops, but there is a tendency for PWS 1 to measure slightly larger and faster drops. For all events, Thies had both the smallest mean drop size and the lowest velocity. However, the mean velocity increased from 1.8 m s^−1^ for events 1–5 to 2.8 m s^−1^ for events 6–8. This may be a result of the wind protection shield installed on the Thies for events 1–5, but not for events 6–8. Thies also recorded a much higher number of drops than the other disdrometers. This could be a result of the design of the Thies disdrometer and drops splashing on the device and thus breaking up into a larger number of smaller drops, as also observed by Angulo-Martínez *et al*. (2018). The larger number of drops measured by Thies may influence the distribution as it is calculated as the percentage of drops out of the total number of drops. If the larger number of small drops is a result of splashing it could be speculated that not all of them are actual raindrops and thus could be removed. This would influence the distributions and increase mean and median drop sizes and velocities.

**Table 3.** Drop size and velocity characteristics of all analysed events.

Event	Device	Total number of drops per m^2^ (10^6^ drops)	% Drops 0–1 mm	% Drops 1–2 mm	% Drops > 2 mm	Mean *D* (mm)	Median *D* (mm)	Mean *V* (m s^−1^)	Median *V* (m s^−1^)
1–5	PWS 1	57.0	66.3	31.2	2.5	0.9 (±0.4)	0.9	3.8 (±1.4)	3.8
	PWS 2	56.3	71.8	26.0	2.2	0.9 (±0.4)	0.8	3.5 (±1.4)	3.4
	Thies	189.7	91.8	7.4	0.7	0.5 (±0.4)	0.3	1.8 (±1.0)	1.6
	Parsivel	72.4	78.5	20.3	1.2	0.8 (±0.4)	0.7	4.1 (±0.8)	4.4
6–8	PWS 2	99.5	78.9	20.4	0.7	0.8 (±0.4)	0.8	3.4 (±1.3)	3.0
	Thies	399.2	94.8	4.9	0.4	0.4 (±0.3)	0.3	2.8 (±1.6)	2.4
	Parsivel	125.8	85.5	14.1	0.4	0.7 (±0.3)	0.7	4.0 (±0.8)	4.4

The mean drop diameter, *D* (± standard deviation) and the mean drop fall velocity, *V* (± standard deviation).

**Figure 7.**
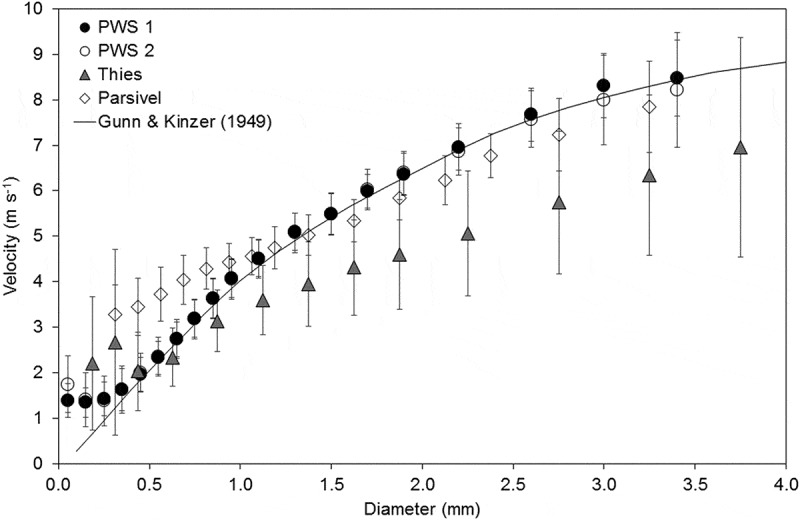
Mean velocity and standard deviation of each drop size class and the terminal fall velocity line drawn after the data by Gunn and Kinzer (1949).

As can be seen from Fig. 7, the mean velocity in each diameter class of the PWS disdrometers follows the terminal fall velocity line of the Gunn and Kinzer (1949) data well, except at the very smallest diameter classes. Except for the smallest drop sizes, Thies underestimated the velocity and had larger standard deviations of the velocity measurements. Parsivel overestimated the velocity of smaller drops up to around 1 mm in diameter, and slightly underestimated the velocity at larger drop diameters. This is consistent with the findings of other studies investigating the first version of Parsivel (Tokay *et al*. 2014, Raupach and Berne 2015). For all disdrometers, the number of drops of the largest drop sizes (from around 3.75 mm) falls below 100 drops and thus the mean velocity was not included for these drop sizes as their reliability is lower. Ellis *et al*. (2006) state that the equations of Best (1950) used for the development of the PWS agree well with the experimental data of Gunn and Kinzer (1949). If the PWS was calibrated to this equation during development, this could explain the close fit of the PWS disdrometers to the terminal fall speed line. Montero-Martínez *et al*. (2016) also found a good fit to the terminal fall speed and a low standard deviation of velocities for the PWS, which they suggested may be due to the removal of drops with anomalous velocities (unknown drops) in the algorithm of the PWS. For Thies, the two lowest diameter classes jumped to a higher mean velocity after the addition of the 2019 events. These events were without the wind protection shield on the Thies disdrometer. In addition, event 6 on 14 March 2019 had the highest mean wind speed of all events (Table 2). It is noteworthy that Thies measured closer to the rain gauge total rainfall in this event than in the other events with lower mean wind speeds. For the 2018 events with the wind protection shield, the mean velocities followed the Gunn and Kinzer line well (results not shown). This may suggest that the wind protection shield is important for estimating the velocity of the smallest drop sizes.

The disdrometers are only capable of separating individual drops into size and velocity classes, which may reduce the precision of the measurements. Taking the mean value of the size and velocity class also introduces an error to the actual size and velocity of the drop. As the width of the size classes increases with increasing drop size and velocity, the error of larger and quicker drops is also increased. Additionally, all values were taken directly from each disdrometer with their individual class widths. This means the drop size and velocity classes are not the same for each disdrometer, which may introduce an error in the comparison of these parameters between the disdrometers. Angulo-Martínez *et al*. (2018) corrected for differences in bin size, which improved consistency, but they still found significant differences between the devices.

**Figure 8.**
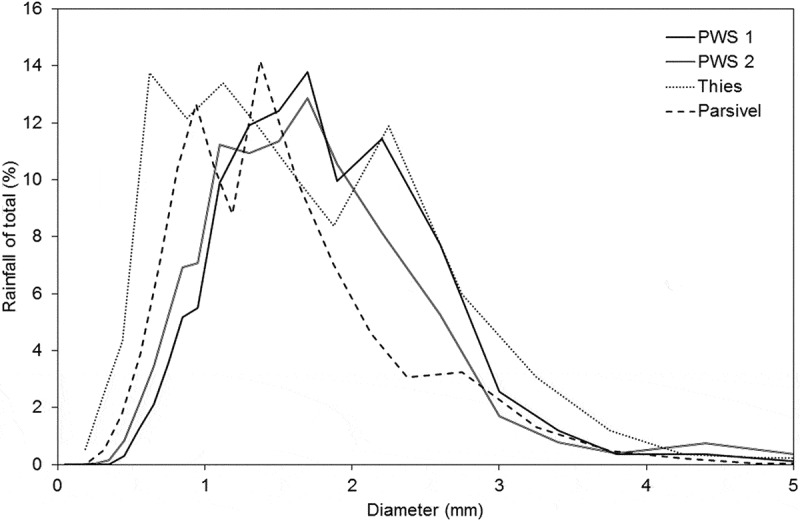
Percentage rainfall out of the total rainfall amount per diameter class for each disdrometer.

Figure 8 shows the percentage of the contribution of each drop size class to the total rainfall. It can be seen, that although the majority of drops were recorded in the range from 0 to 1 mm in diameter (Fig. 6), these drops did not make up the majority of the fallen rain. Even though the very high number of small drops in the two smallest size classes accounted for more than 50% of the drops measured by the Thies disdrometer, their size limits the rainfall associated with them to 3% of the total amount. The majority of rainfall fell as drops of 1–2 mm in diameter. For PWS 1, PWS 2, Thies and Parsivel these drops made up 58%, 57%, 44% and 51% of the total rainfall, respectively. A smaller percentage of rainfall was attributed to drop sizes between 2 and 3 mm for Parsivel compared to the other disdrometers. Parsivel assigns 11% of the total rainfall to these drop sizes, whereas the percentages of the other disdrometers range between 15% and 22%. As this rainfall is calculated from the DSD, it is different from the rainfall amount shown per event, which was taken from the disdrometer output. The differences in drop size estimation between the disdrometers clearly affect their differences in total rainfall.

### 3.3 Kinetic energy

Drop size and velocity are also crucial for estimation of kinetic energy. PWS 1 measured the highest total KE per event (Table 4) due to the largest mean drop size and velocity (with the exception of Parsivel). PWS 2 measured lower total KE per event than PWS 1 with a relative bias of −14.5%. However, the total KE per mm rainfall of the analysed events does almost not differ at all between the two PWS. This fits well with PWS 2 measuring less total rainfall than PWS 1, but both of them having similar drop size and velocity distributions, which leads to similar KE per mm rainfall. Even though Parsivel measured less accumulated rainfall for all but two events, it still has a higher total KE for all the events in 2018 than Thies. This is a result of the small mean drop size and low velocity measured by Thies, which leads to a lower KE. For the events in 2019 Thies measured higher total KE than Parsivel. This may be a result of the altered velocity of the smallest drop sizes, as the wind protection shield had been removed. The relative bias of KE per mm rainfall of Thies and Parsivel was −26% and −13% compared to the PWS 2 disdrometer. Angulo-Martínez and Barros (2015) found a difference of 2–3% in cumulative KE between Parsivel disdrometers (versions 1 and 2) at one site, whereas the difference in cumulative KE was 43% at another site. The large variation in measurement difference was attributed to the difference in DSD between the two sites.

**Table 4.** Total kinetic energy (KE) and kinetic energy per mm of rainfall measured by each disdrometer for the selected events.

Device	Event 1		Event 2		Event 3		Event 4		Event 5		Event 6		Event 7		Event 8	
	01 September 2018		04 September 2018		21 September 2018		23 September 2018		01 October 2018		14 March 2019		30 April 2019		03 May 2019	
	KE		KE		KE		KE		KE		KE		KE		KE	
	(J m^−2^)	(J m^−2^ mm^−1^)	(J m^−2^)	(J m^−2^ mm^−1^)	(J m^−2^)	(J m^−2^ mm^−1^)	(J m^−2^)	(J m^−2^ mm^−1^)	(J m^−2^)	(J m^−2^ mm^−1^)	(J m^−2^)	(J m^−2^ mm^−1^)	(J m^−2^)	(J m^−2^ mm^−1^)	(J m^−2^)	(J m^−2^ mm^−1^)
PWS 1	327.3	16.7	105.4	16.9	107.7	16.8	92.4	15.7	122.4	13.0	-	-	-	-	-	-
PWS 2	288.2	16.7	88.7	16.9	81.7	16.7	79.0	15.7	115.7	13.2	192.1	10.2	320.9	15.0	139.6	13.7
Thies	187.5	11.7	58.3	12.3	59.7	13.4	42.3	10.7	59.7	8.3	176.3	8.3	230.5	12.8	100.2	10.3
Parsivel	210.1	14.6	65.5	14.3	75.0	15.5	56.9	13.5	81.1	11.5	166.1	11.0	204.9	12.4	96.1	11.8

**Figure 9.**
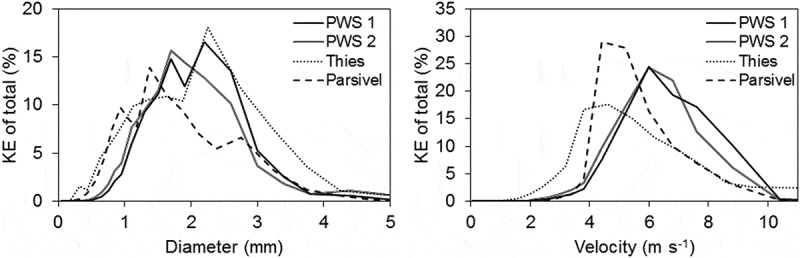
Percentage kinetic energy per drop size (left) and velocity class (right) for each disdrometer.

The distribution of KE per drop size and velocity class is presented in Fig. 9. PWS 1 and 2 have similar distributions of KE per drop size and Thies does not deviate much from this distribution, whereas Parsivel is more left-skewed. This shows that even though Thies has quite a different drop size distribution from the two PWS, the KE distribution is similar, as the small drops do not contribute much to KE. The majority of the KE for PWS 1 and 2 and Parsivel is due to drops between 1 and 2 mm. These drop sizes contribute 53%, 59% and 51% of the total KE for PWS 1, PWS 2 and Parsivel, respectively. The total KE measured by Thies is more evenly distributed between the drop size classes between 1–2 mm and 2–3 mm with 42% and 41% each.

As seen from the results above, the same rainfall measured with different instruments results in different drop size and velocity distributions. The device-specific drop size and velocity measurements influence the KE–*I* relationships and make it difficult to compare KE from different disdrometers. This may affect the estimation of rainfall erosivity, which rely on measurements of intensity and KE. In addition, spatial variations in rainfall characteristics can only be quantified if measurements from different devices are comparable. We therefore propose a correction factor (CF) for KE, which enables the direct comparison of KE between devices.

**Figure 10.**
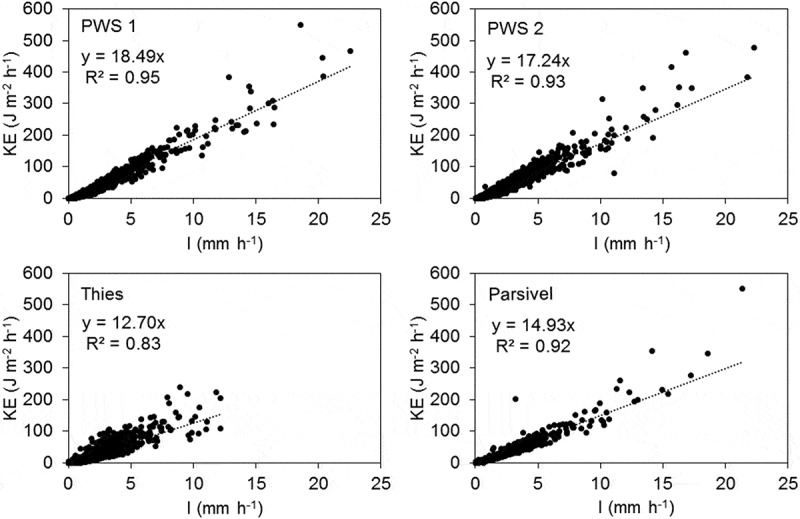
Kinetic energy versus rainfall intensity for all rainy minutes of the selected events with linear regression for each disdrometer.

Figure 10 shows the relationship between intensity and KE for each disdrometer for all rainy minutes recorded during the analysed events. The maximum minute intensities recorded were 22.5, 22.3, 12.1 and 21.3 mm h^−1^ for PWS 1, PWS 2, Thies and Parsivel, respectively. As the PWS disdrometers had the best agreement with the rain gauge and measured similar drop size and velocity distributions, and PWS 2 measurements were available during the entire study period, PWS 2 was chosen as the reference for the Thies and Parsivel disdrometers. A linear regression was fitted to the KE–*I* relationships of each disdrometer. The linear regression line was forced through the origin and equations with a slope coefficient, *β*, was found for each disdrometer. The ratio of slope coefficients of PWS 2 on the one hand, and that of Thies or Parsivel on the other hand gives correction factors for the two devices relative to PWS 2:

(5) $$CF_{Thies}=\frac{\beta_{PWS2}}{\beta_{Thies}}$$

(6) $$CF_{Parsivel}=\frac{\beta_{PWS2}}{\beta_{Parsivel}}$$

**Figure 11.**
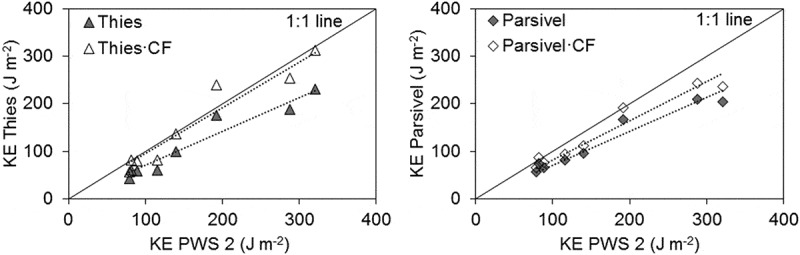
Kinetic energy of each event measured by PWS 2 as compared to Thies (left) and Parsivel (right) and the event KE multiplied with the correction factor (CF) of 1.36 for Thies and 1.15 for Parsivel.

By using Equations (5) and (6), the correction factor was found to be 1.36 for Thies and 1.15 for Parsivel. Applying this correction factor to the KE of each event led to KE values that were closer to those measured by PWS 2 (Fig. 11). The bias of the total KE for all eight events measured by Thies decreased from –48.9 to –8.1 J m^−2^ and for KE per mm rainfall it decreased from –3.8 to 0.1 J m^−2^ mm^−1^. For Parsivel application of the correction factor reduced the bias of total KE of all events from –43.8 to –25.3 J m^−2^ and of KE per mm rainfall from –1.7 to 0.3 J m^−2^ mm^−1^.

To increase the accuracy of the correction factor, the linear regression of the KE–*I* relationship could be applied to each single event. When doing so for the eight events investigated, the correction factor ranges between 1.16 to 1.52 and 0.98 to 1.25 for Thies and Parsivel, respectively. However, we consider the average CF of all eight events to be generally applicable. Using a simple correction factor for KE is useful when one is interested in KE. However, it is also possible to correct for the differences in drop size distribution and velocity between instruments before calculating KE. Other studies have used theoretical models for calculating terminal fall velocity from the drop sizes instead of using the velocity estimated by the disdrometers (Jaffrain and Berne 2012, Angulo-Martínez and Barros 2015). This eliminates one uncertainty between multiple devices, as one parameter is calculated the same way. It may help correct for any difference in velocity measurements between devices, but it might also remove some actual differences between devices. Petan *et al*. (2010) found a large dispersion of the KE–*I* relationship for a Thies disdrometer. The dispersion decreased after using a theoretical fall velocity model before calculation of KE, and it resulted in an average increase of 6% in event kinetic energy. However, this method does not correct the drop size distribution. Raupach and Berne (2015) corrected drop concentration per size class of Parsivel disdrometers using the measurements of a 2DVD disdrometer as a reference. The extensive filtering and data processing needed for the 2DVD disdrometer may not be feasible for the average user. On the other hand, as can be seen from previous studies as well as the present one, there are significant differences between instruments, which must be considered in the estimation of kinetic energy.

## 4 Conclusions

This study compared three types of optical laser-based disdrometers collocated with a weighing rain gauge under natural rainfall conditions. The disdrometers were found to underestimate total rainfall compared to the rain gauge. Due to differences in drop size and velocity, the rainfall KE differed between instruments. A correction of KE between devices was introduced based on a linear regression of the KE–*I* relationship of each disdrometer. Applying the device-specific correction factor to the KE of each event greatly reduced the differences between the disdrometers.

The differences in total measured rainfall were found to relate to the design and internal data processing of each disdrometer. However, as the internal data processing is not fully known to the user, these differences cannot be fully corrected. To ensure accurate estimation of drop size and velocity there is still a need for further improvement of disdrometers and the processing of their data. Our focus was not on the correction of the differences in DSD and velocity of the disdrometers, although this has been attempted in other studies, but on the correction of KE for a more consistent comparison of devices. Detailed knowledge of the measurement differences between disdrometers allows for comparison of rainfall characteristics of multiple disdrometer types at one or more sites. Extended measurement series with a large range of rainfall events would provide more complete information on rainfall characteristics and could be used to examine site-specific conditions, spatiotemporal variations and other effects such as wind. As all instruments are associated with some error, an intercomparison of disdrometer types is necessary to quantify differences between devices and provide more robust results.
